# Therapeutic Management of Gastroesophageal Reflux Disease (GERD)—Is There Something Between PPI and Fundoplication? An Overview

**DOI:** 10.3390/jcm14020362

**Published:** 2025-01-09

**Authors:** Harald Rosen, Christian Sebesta, Marie Christine Sebesta, Christian Sebesta

**Affiliations:** 1 Centre of Surgery, Sigmund Freud Private University, A-1020 Vienna, Austria; rosensurg@compuserve.com; 2 Department of Gastroenterology, Clinic Donaustadt, SMZ-Ost, Langobardenstrasse 122, A-1220 Vienna, Austria; 3 Department of Internal Medicine, Clinic Donaustadt, SMZ-Ost, Langobardenstrasse 122, A-1220 Vienna, Austria

**Keywords:** gastroesophageal reflux, endoscopy, laparoscopy, transoral procedures

## Abstract

Gastroesophageal reflux disease (GERD) affects millions globally, with traditional treatments like proton pump inhibitors (PPIs) and surgical fundoplication presenting challenges such as long-term medication dependency and disturbing long term side effects following surgery. This review explores emerging, alternative therapies that offer less invasive, personalized alternatives for GERD management. Endoscopic approaches, including Stretta therapy, transoral incisionless fundoplication (TIF), and endoscopic full-thickness plication (EFTP), demonstrate promising but also controversial outcomes in symptom relief and reduced acid exposure. Laparoscopic electrical stimulation therapy (EndoStim^®^) and the LINX^®^ magnetic sphincter augmentation system address LES dysfunction, while endoscopic anti-reflux mucosectomy and/or ablation techniques aim to construct a sufficient acid barrier. The RefluxStop™ device offers structural solutions to GERD pathophysiology with intriguing results in initial studies. Despite promising results, further research is required to establish long-term efficacy, safety, and optimal patient selection criteria for these novel interventions. This review underscores the importance of integrating emerging therapies into a tailored, multidisciplinary approach to GERD treatment.

## 1. Introduction

Gastroesophageal reflux disease (GERD) is a prevalent condition affecting millions globally, characterized by the regurgitation of stomach contents into the esophagus, leading to a spectrum of symptoms that can significantly impair quality of life [1].

The traditional management of GERD has primarily relied on proton pump inhibitors (PPIs), which effectively reduce gastric acid production and alleviate symptoms for many patients [2]. However, the long-term reliance on PPIs raises concerns regarding potential side effects and complications, prompting the search for alternative therapeutic options. Laparoscopic fundoplication has proven effective in providing lasting relief but is often reserved for patients with severe or refractory cases due to their invasive nature and associated side effects like dysphagia and/or bloating symptoms [3,4].

This review explores the evolving landscape of GERD management, investigating the potential therapeutic strategies that lie between these two therapeutic standards: PPIs and surgical fundoplication.

By examining novel minimally invasive procedures, we aim to identify effective approaches that can bridge the gap between conservative management and surgical intervention, ultimately enhancing patient outcomes in the management of GERD.

The evaluation of the patients in the studies cited in this paper followed different diagnostic algorithms, whereby gastroscopy and esophageal manometry were essential prerequisites for the allocation before any kind of interventional treatment or surgery.

New strategies can be divided into endoscopically or laparoscopically performed approaches (Table 1).

## 2. Stretta Therapy: Radiofrequency Ablation for the Lower Esophageal Sphincter (LES)

The Stretta procedure is a non-surgical, minimally invasive treatment for GERD that was been approved by the FDA in 2000 and has been applied in more than 25,000 patients worldwide [2,5,6]. It uses radiofrequency energy to strengthen the LES, aiming to reduce acid reflux. Stretta involves delivering controlled radiofrequency energy to the muscle tissues of the LES and gastric cardia, inducing structural changes intended to enhance LES function. These changes may include collagen deposition, fibrosis of submucosal layers, and hypertrophy of the muscular layer, all contributing to a more robust LES barrier against reflux [7]. This mechanism theoretically reduces the frequency of transient LES relaxations (TLESRs), which are a primary cause of GERD symptoms.

While the precise mechanisms by which the Stretta procedure exerts its effects are not fully understood, several theories have been proposed. One hypothesis suggests that RF energy induces a controlled form of coagulative necrosis in the tissue, leading to subsequent healing through fibrosis [2]. However, given that the mucosal temperature is maintained well below the threshold for tissue ablation (100 °C), significant tissue destruction is unlikely to occur.

Alternatively, some studies have indicated that the Stretta procedure may influence the neuromuscular function of the LES [8]. Research involving pigs demonstrated that Stretta significantly restored LES pressure following a botulinum toxin injection, with notable increases in gastric yield pressure in the Stretta-treated group compared to controls, suggesting that Stretta can reverse some of the effects induced by the toxin [8]. Additionally, a study focusing on dogs found that applying Stretta specifically to the gastric cardia could prevent the triggering of transient lower esophageal sphincter relaxations (TLESRs), which are often responsible for gastroesophageal reflux [9].

In a randomized crossover study, Arts et al. examined the effects of Stretta on gastroesophageal junction (GEJ) resistance, concluding that the procedure decreased GEJ compliance, potentially decreasing the volume of refluxate and contributing to symptom relief [10]. Supporting this, Perry et al. noted that radiofrequency energy delivery to the LES can reduce esophageal acid exposure while improving GERD symptoms [11]. Based on these observations, it was assumed that the treatment directed at the gastric cardia may play a crucial role in the mechanism of action for Stretta. By directly applying RF energy to the sling and clasp fibers of the gastric cardia, the procedure may effectively reduce tissue compliance and thereby diminish TLESRs, leading to improved outcomes for patients with GERD [2].

### Clinical Evidence and Outcomes

Several clinical trials and meta-analyses have evaluated the effectiveness of Stretta therapy for GERD. For instance, a meta-analysis by Perry et al. reviewed data from 1441 patients across 18 studies, revealing that Stretta significantly improved GERD-specific quality of life as measured by heartburn scores (*p* = 0.001) and the GERD-health-related quality-of-life (HRQL) scale (*p* = 0.001) [11]. The study further reported a notable decrease in esophageal acid exposure, evidenced by a reduction in Johnson–DeMeester scores from 44.4 to 28.5 (*p* = 0.007), indicating a positive impact of Stretta on acid exposure.

In accordance with this, in a randomized, sham-controlled trial by Corley et al. (2003), participants who underwent Stretta experienced notable enhancements in GERD symptoms compared to those receiving sham treatment, reinforcing the procedure’s efficacy in symptomatic relief [12].

Additional studies have further corroborated these findings [6,12,13,14,15,16]. Liu et al. (2011) reported substantial improvements in clinical parameters following radiofrequency energy delivery, indicating that patients typically experience relief from troublesome symptoms [13]. The research of Coron et al. (2008) specifically focused on patients reliant on proton pump inhibitors (PPIs), demonstrating that radiofrequency energy delivery could effectively reduce dependence on these medications, offering a viable alternative for those seeking to minimize long-term PPI use [14].

Long-term follow-up studies have also emphasized the durability of the Stretta procedure. For instance, Dughera et al. (2011) conducted a prospective study that reported sustained symptom improvement over a 48-month period [15], while Triadafilopoulos et al. (2002) documented positive outcomes at both 6 and 12 months in an open-label trial [6]. Noar and Lotfi-Emran (2007) extended this evidence, showing continued symptom relief and reduced antisecretory drug use four years post-procedure [16].

Moreover, the research conducted by Reymunde and Santiago (2007) confirmed long-term benefits, including improvements in quality of life and medication usage after radiofrequency energy delivery for GERD [17]. An 8-year follow-up study by Dughera et al. (2014) further established the lasting effects of the Stretta procedure, suggesting that its benefits (improvement in QOL, no need for PPI medication) can endure well beyond the initial treatment period [18].

However, the procedure’s effectiveness remains a topic of debate within the clinical community due to mixed evidence. A systematic review by Lipka et al. found that Stretta, when compared to sham treatments, did not produce substantial changes in physiological parameters like time spent with a pH below 4, LES pressure, or the ability to discontinue PPIs [19]. This review argued that the therapeutic benefits of Stretta may not be as pronounced as initially thought, especially in physiological measurements critical to GERD symptom management.

Professional organizations and guidelines provide additional perspectives on Stretta’s place in GERD management. The American College of Gastroenterology’s (ACG) 2022 clinical guidelines note the inconsistent and highly variable outcomes of radiofrequency energy treatments like Stretta, advising against its routine use as an alternative to medical or surgical therapies [20].

This is recommendation is in strict contrast to the 2013 guidelines of the Society of American Gastrointestinal and Endoscopic Surgeons (SAGES), which recommend Stretta for patients who prefer to avoid traditional fundoplication, though they advise caution due to variable efficacy data [2].

Given these findings, Stretta may be a viable treatment option for a subset of GERD patients who either do not respond well to PPIs or seek to avoid surgery. However, further studies are needed to better understand which patient populations may benefit most from this approach.

## 3. Transoral Incisionless Fundoplication (TIF) and Endoscopic Full Thickness Plication (EFTP)

Transoral incisionless fundoplication (TIF) and endoscopic full-thickness plication (EFTP) are both endoscopic procedures used to treat gastroesophageal reflux disease, but they differ in techniques, target anatomy, and procedural goals.

### Procedure Technique

- TIF: TIF is typically performed using the EsophyX (Endogastric Solutions, Merit Medical, UT, USA)) device, which uses fasteners to create folds in the stomach at the gastroesophageal junction. The goal is to reconstruct the valve-like function of the lower esophageal sphincter (LES) by creating a tighter anti-reflux barrier. TIF involves the creation of a 270–300° fundoplication, making it less invasive than traditional fundoplication surgery.
- EFTP: EFTP, by contrast, involves the use of transmural sutures, which go through the entire thickness of the stomach wall to secure the plications. This technique aims to improve LES function by placing full-thickness sutures at specific points to bolster the gastroesophageal junction’s barrier. Because it penetrates deeper layers of the tissue, EFTP can offer a more robust structural alteration. Endoscopic full-thickness plication is performed using the GERDx™ system (G-SURG GmbH, Seeon-Seebruck, Germany). The GERDx™ system is the advanced single use product of a company that has taken over the Plicator technology, after the Plicator device (Ethicon Endosurgery, Sommerville, NJ, USA) was taken off the market

## 4. Efficacy and Symptom Relief

### 4.1. TIF

The efficacy of transoral incisionless fundoplication (TIF) for treating GERD symptoms has been demonstrated in several clinical studies, with concrete outcome measures highlighting its benefits.

In the TEMPO trial, approximately 88% of patients were able to eliminate or significantly reduce their use of PPIs at one-year post-procedure [21,22].

Another meta-analysis of randomized trials by Gerson et al. confirmed that TIF improved GERD symptoms, with 70–80% of patients experiencing a significant reduction in heartburn and regurgitation compared to medical therapy alone [23].

Hunter et al. showed that TIF significantly decreased distal esophageal acid exposure, with 85% of patients achieving normalized pH levels, as opposed to only 12% in the PPI group. Patients in the TIF group also reported improved quality of life scores [24].

Rinsma et al. reported that TIF was effective in increasing lower esophageal sphincter (LES) resting pressure, which is critical for reducing reflux events [25]. Their findings indicated an improvement in LES function, with significant reductions in reflux episodes measured by impedance-pH monitoring.

Barnes et al. found high satisfaction rates with TIF among patients, with over 80% reporting improved symptoms and quality of life. Additionally, 67% of patients expressed satisfaction with their ability to stop or reduce medication post-TIF [26].

Another study by Bell et al. reported that 76% of patients achieved clinically significant improvement in GERD symptoms and noted enhanced quality of life following TIF [27].

### 4.2. Long-Term Efficacy

Longitudinal data from a cohort study by Chimukangara et al. indicated that 74% of patients continued to experience symptom relief up to eight years post-TIF [28].

Testoni et al. documented sustained symptomatic relief and a decrease in PPI dependency in 65–75% of patients between three and ten years after TIF [29].

The TEMPO trial showed a 72% reduction in regurgitation symptoms and 67% reduction in extraesophageal symptoms at five-year follow-up [22].

## 5. Safety

Transoral incisionless fundoplication (TIF) is generally considered safe, but the literature does detail some specific complication rates to consider.

Mild or moderate pain, including sore throat, is common immediately after TIF and typically resolves within days to weeks. According to Trad et al. in the TEMPO trial, around 40% of patients reported transient sore throat and abdominal pain, which resolved without intervention [21].

Dysphagia, or difficulty swallowing, is reported in 3–8% of patients. Bell et al. found that 4% of patients experienced dysphagia post-TIF, generally resolving within a few weeks. In rare cases, dysphagia may persist, necessitating further treatment [27].

Postoperative bloating and flatulence were reported by approximately 15–20% of TIF patients in various studies, often associated with temporary alterations in gastric emptying or the restructured gastroesophageal junction. This was observed in a study by Gerson et al., where around 17% of patients reported bloating symptoms that tended to diminish over time [23].

While rare, perforation remains a serious potential complication. The rate of esophageal or gastric perforation is reported at less than 0.5% [23,30]. These cases often require surgical intervention.

Minor bleeding occurs in 1–2% of patients and is usually managed conservatively. In the TEMPO trial, bleeding was documented in about 1.3% of patients and typically did not require additional procedures [21].

## 6. EFTP 

**Figure 1:**
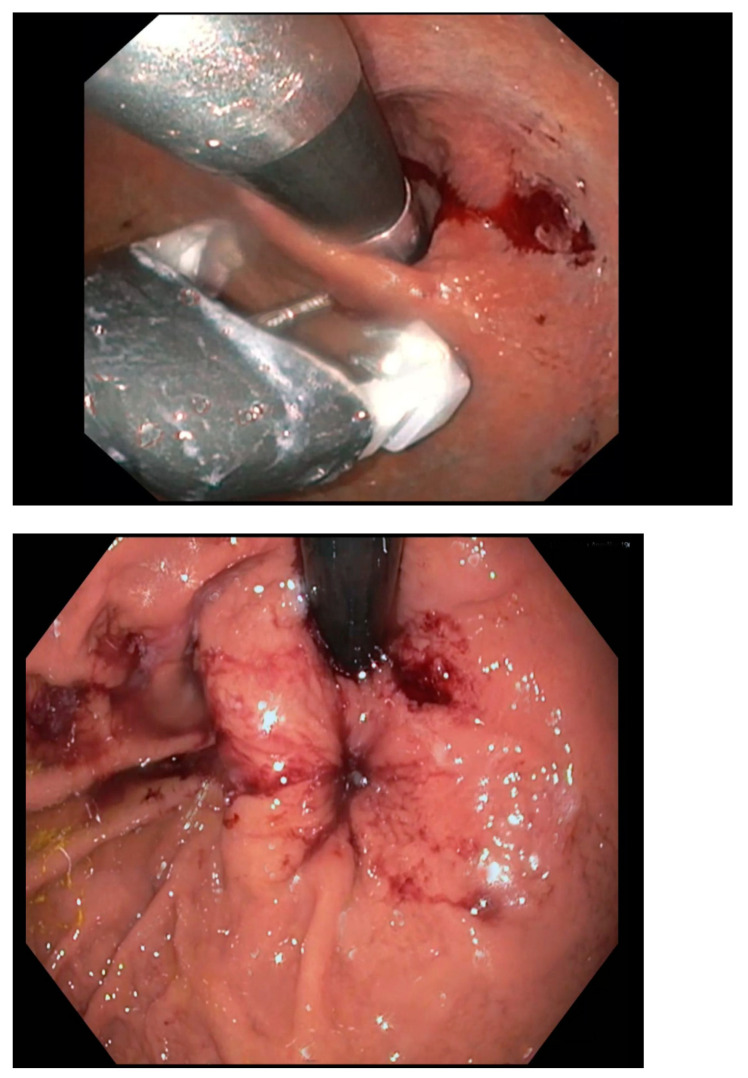
Endoscopic Full Thickness Plication (EFTP).

Endoscopic full-thickness plication (EFTP) has shown promising efficacy in treating GERD (Figure 1), with multiple studies reporting symptom relief, improved quality of life, and reduced dependence on medication.

A meta-analysis by Gerson et al. reported that around 60–70% of GERD patients experienced significant symptom relief following EFTP [23]. Patients showed marked reductions in heartburn, regurgitation, and overall GERD-related symptoms within the first year of follow-up.

Pleskow et al. observed a reduction in GERD symptoms in nearly 80% of patients six months after EFTP, highlighting its potential to address both typical and atypical GERD symptoms effectively [31].

Other studies suggest that EFTP significantly reduces the need for daily PPIs [32,33]. According to von Renteln, about 70% of patients who underwent EFTP reported either complete cessation or reduced PPI usage by at least 50% within one-year post-procedure [34].

EFTP has also been shown to improve esophageal acid exposure, an objective measure of GERD severity [32]. Studies with follow-up periods of one to two years generally report improvements in GERD symptoms, such as heartburn and regurgitation, as well as reductions in the need for proton pump inhibitor (PPI) medications.

Kalapala et al. conducted a study that observed the effects of GERD-X over 12 months and noted sustained symptom relief in a significant portion of patients [33]. They reported reductions in PPI usage and symptom improvement rates close to 60–70% at the one-year follow-up.

Another study by Kaindlstorfer et al. showed that up to 60% of patients reported continued symptom improvement and quality of life enhancements three years after undergoing EFTP, although some did experience mild symptom recurrence that was manageable with occasional medication [35].

EFTP appears to strengthen the lower esophageal sphincter (LES) by creating full-thickness folds, which improve its pressure and function. Studies like those reviewed by Niu et al. show that EFTP has led to improved LES pressure in patients with weakened sphincter function, which correlates with better symptom control and less reflux [36].

EFTP may require further studies to determine its durability and effectiveness over more extended periods (e.g., 5–10 years) as there is currently a gap in the literature for long term follow-up.

## 7. Combined Transoral and Laparoscopic Approach (c-TIF)

The approach of performing concomitant laparoscopic hiatal hernia (HH) repair with transoral incisionless fundoplication (TIF) has gained traction as an interdisciplinary strategy for managing GERD, particularly in patients with larger hiatal hernias. This combined procedure seeks to improve symptom relief by addressing both the anatomical defect at the hiatus and the weak anti-reflux barrier at the gastroesophageal junction.

In a study by Choi et al., 85% of patients who underwent this combined procedure experienced significant reduction or complete resolution of GERD symptoms, along with a 71% decrease in daily PPI use [37]. Additionally, quality of life improved markedly, with significant reductions in both heartburn and regurgitation scores, which remained stable at the one-year follow-up. Objective measures, such as esophageal pH monitoring, revealed that acid exposure dropped substantially in patients following the procedure.

Supporting these findings, Ihde reported similar results, noting that patients had a 50–60% reduction in acid exposure time (AET) after undergoing the combined HH repair and TIF [38]. Additionally, around 76% of patients achieved normalized AET, which reflects a substantial improvement in acid control. This objective success aligns with self-reported outcomes, where over 80% of participants reported symptom relief and high satisfaction

Bazerbachi et al. reviewed data showing that the combined procedure provides greater durability of symptom control compared to TIF alone, especially in patients with larger hiatal hernias. The combined technique not only reinforced the GEJ but also significantly reduced the risk of hernia recurrence, with less than 10% of patients experiencing symptomatic recurrence over the one-year period [39].

Janu et al. examined the effectiveness of the procedure across two community hospitals [40]. They observed a 60% reduction in symptom severity scores and a 75% reduction in daily PPI dependency, demonstrating the practicality and efficacy of this approach outside of specialized centers. Furthermore, approximately 85% of patients reported satisfaction with their GERD symptom management following the combined procedure [40].

Overall, combining HH repair with TIF has shown significant and sustained efficacy in reducing GERD symptoms, acid exposure [41], and medication dependency, with high patient satisfaction and symptom control lasting up to one year or more post-procedure.

## 8. Transoral Antegrade Suturing Devices

The OverStitch device (Apollo Endosurgery, Austin, TX, USA) is an advanced full-thickness endoscopic suturing system designed to enable endoscopists to perform suturing in various areas of the gastrointestinal tract [42]. Primarily, it has been utilized in the management of GERD by augmenting the gastroesophageal junction (GEJ) to improve the anti-reflux barrier, as well as in closing fistulas, managing perforations, and reducing the volume of the stomach for weight loss procedures [43] 

Furthermore, Banerjee et al. reviewed various endoscopic closure devices, including OverStitch, noting that its design allows for precision and control in placing sutures, which is critical for procedures that require full-thickness tissue apposition [44]. They highlighted the versatility of OverStitch in both therapeutic and surgical endoscopy, with applications extending from gastrointestinal closure and reconstruction to bariatric interventions. The device is praised for its ability to create continuous or interrupted sutures, providing endoscopists with flexibility depending on the clinical scenario [44].

Han et al. explored the efficacy of the OverStitch device specifically for GERD patients, demonstrating that it allows for full-thickness plications at the GEJ, which enhance the anti-reflux barrier [45]. In their study, patients showed symptomatic improvement in GERD-related symptoms post-procedure, as well as reductions in PPI usage [45]. The ability of OverStitch to create robust, durable sutures provides an advantage over other endoscopic devices, as it offers stability and may decrease the likelihood of recurrence compared to superficial plications.

In summary, the OverStitch device represents an effective tool for reinforcing the GEJ in GERD patients, with studies supporting its efficacy in reducing symptoms and medication reliance. Additionally, its versatility in endoscopic interventions makes it a valuable asset for a range of therapeutic applications, as well as an additional tool to be used for mucosal resection procedures, which will be dealt with in the following sections.

## 9. Endoscopic Resection/Ablation Techniques (Table 2)

Endoscopic resection and ablation techniques offer innovative options for patients with gastroesophageal reflux disease (GERD), especially those who do not respond to proton pump inhibitors (PPIs) or seek non-surgical interventions. These approaches, which include anti-reflux mucosectomy (ARMS), mucosal ablation and suturing (MASE), and resection and plication (RAP), focus on modifying the gastroesophageal junction to reduce acid reflux effectively.

**Table 2:** Endoscopic resection/ablation techniques.

ARMS	Anti-Reflux Mucosectomy
MASE	Mucosal Ablation and Suturing
ARMS with Band Ligation	Banded Anti-Reflux Mucosectomy
RAP	Resection and Plication

## 10. Anti-Reflux Mucosectomy (ARMS)

Anti-reflux mucosectomy (ARMS) involves resecting mucosal tissue around the gastroesophageal junction to induce fibrosis, which can help strengthen the anti-reflux barrier. In a pilot study by Inoue et al., 68% of GERD patients without a hiatal hernia showed a significant reduction in acid exposure and symptom scores post-ARMS [46]. In another study by Patil et al., ARMS yielded promising results in the first Indian cohort [47], where 70% of patients achieved sustained symptom relief and were able to discontinue PPIs six months after the procedure.

In a recent publication, Ota et al. noted that up to 85% of patients who underwent ARMS remained symptom-free and off PPIs at two-year follow-up, demonstrating both short-term efficacy and potential long-term durability [48].

Further innovations in ARMS involve cap-assisted endoscopic mucosal resection to enhance accuracy and safety [49]. Lee et al. studied this method in a cohort with refractory GERD and found that 74% of patients experienced significant symptom improvement, with effects lasting up to two years [49]. Zhu and Shen also highlighted that ARMS can yield a stable reduction in GERD symptoms and acid exposure, indicating its potential for durable outcomes in PPI-dependent GERD cases [50].

## 11. Mucosal Ablation and Suturing (MASE)

MASE combines mucosal ablation with suturing at the gastroesophageal junction, aiming to tighten the lower esophageal sphincter (LES) area. In the study by Han et al., patients undergoing MASE reported an 80% improvement in GERD symptoms, as measured by GERD-HRQL (Health-Related Quality of Life) scores, and a significant reduction in acid exposure at one-year follow-up [45]. Most patients were able to reduce or stop their PPI use, with 85% reporting satisfaction with the procedure.

## 12. Banded Anti-Reflux Mucosectomy (ARMS with Band Ligation)

The banded ARMS approach uses band ligation to facilitate a controlled resection, aiming to further reduce acid reflux risk and improve symptom outcomes. Hedberg et al. (2019) introduced this technique and reported a 75% reduction in heartburn symptoms with minimal adverse events in their cohort [51]. Similarly, Monino et al. (2019) found that band-assisted ARMS was associated with high patient satisfaction and effective symptom control in refractory GERD cases [52]. Using band ligation, these studies show that controlled resection can improve outcomes with reduced complications, making it a viable option for patients with limited alternatives [51,52].

## 13. Resection and Plication (RAP)

The resection and plication (RAP) technique combines mucosal resection with plication to create a tighter gastroesophageal junction, preventing acid backflow. In a study by Benias et al., RAP led to a 70% reduction in GERD symptoms among patients, with 67% showing marked improvement in quality-of-life scores related to reflux [53]. Importantly, this method allows for more comprehensive remodeling of the gastroesophageal junction

## 14. Safety of Resection/Ablation Techniques

Techniques like anti-reflux mucosectomy (ARMS), mucosal ablation and suturing (MASE), and resection and plication (RAP) have demonstrated a good safety profile but are associated with potential complications. Common issues reported in the literature include postoperative pain, bleeding, dysphagia, and esophageal strictures, with complication rates varying depending on the specific technique and patient population:

1. Bleeding: Bleeding is a notable risk, particularly with mucosal resection techniques. Studies such as those by Monino et al. [51] and Lee et al. [49] report minor to moderate bleeding in approximately 5–10% of patients following procedures like ARMS. In most cases, bleeding is managed endoscopically without the need for further intervention.
2. Esophageal Stricture: Esophageal stricture, a narrowing of the esophagus, is a less frequent but significant complication. This complication occurs in about 1–5% of patients after ARMS procedures, according to research by Zhu and Shen [50]. Patients who develop strictures often require follow-up dilations to alleviate symptoms of dysphagia. Lee et al. [49] reported that in their cohort, around 5–10% of patients who underwent ARMS required esophageal dilation to address stricture-related dysphagia. These dilations were often necessary within the first few weeks post-procedure. In a study by Ota et al. on ARMS with cap-assisted endoscopic mucosal resection, about 5% of patients developed strictures that needed endoscopic dilation, typically with satisfactory resolution after one or two dilation sessions [54]. Patil et al. also noted that while strictures are an infrequent complication, they occasionally require one or more dilations to alleviate symptoms, especially in patients who underwent larger resections [47]. The dilation procedures generally have good outcomes, with most patients experiencing a resolution of dysphagia after a few sessions. However, the need for dilation tends to be more common in patients who undergo extensive mucosal resections, underscoring the importance of procedural techniques in minimizing such complications [47]. These findings highlight the value of follow-up care to manage complications effectively in endoscopic GERD therapies.
3. Dysphagia: Dysphagia is reported by up to 10–15% of patients in the short-term, though it typically resolves over time. In Hedberg et al.’s study of banded ARMS, approximately 12% of patients experienced dysphagia initially, but symptoms generally subsided within a few weeks [51].
4. Perforation: Perforation is a rare but serious complication requiring surgery, occurring in less than 1% of cases, as reported in studies by Han et al. [45] and Ota et al. [54].

Endoscopic techniques such as ARMS, MASE, and RAP offer promising minimally invasive options for managing GERD. They provide significant symptom relief, often enabling patients to reduce or eliminate PPI use. However, these procedures are offered by institutions specialized in interventional endoscopy and more data and longer follow-up will be required to establish their precise role in the therapeutic algorithm.

## 15. Laparoscopic Techniques

### Magnetic Sphincter Augmentation

**Figure 2:**
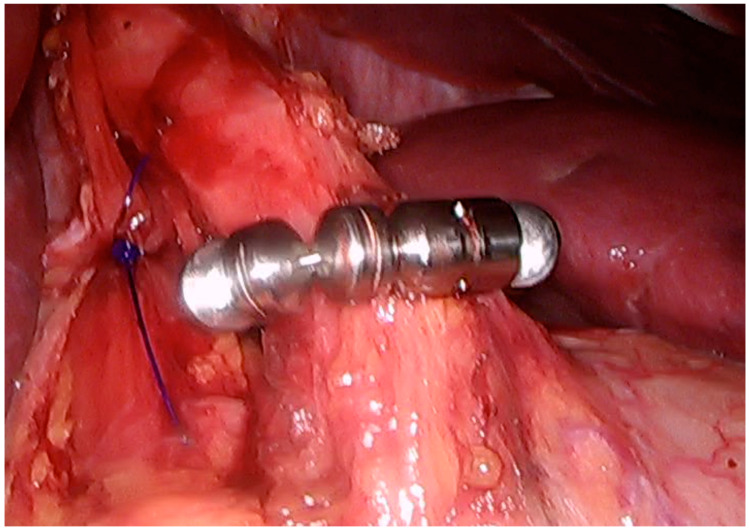
LINX ring implanted at the distal esophagus.

The LINX magnetic sphincter augmentation (MSA) device is a ring of magnetic beads placed around the lower esophageal sphincter (LES) designed to reinforce the LES, reducing acid reflux by preventing gastric contents from moving back into the esophagus while still allowing normal swallowing and burping (Figure 2).

The LINX ring shares some conceptual similarities with earlier surgical innovations, such as the Angelchik prosthesis, developed by Angelchik and Cohen in 1979 [55]. The Angelchik device, a C-shaped silicone ring positioned around the lower esophageal sphincter (LES), was intended to reinforce the LES and reduce acid reflux, much like the LINX device. However, this device has encountered challenges, particularly with device erosion and migration, which have affected their long-term success and safety profiles [56].

The LINX magnetic sphincter augmentation (MSA) device has emerged as a promising intervention for managing gastroesophageal reflux disease (GERD), providing a less invasive alternative to traditional fundoplication surgeries. The data from numerous studies illustrate both the effectiveness and complications associated with LINX [57,58,59,60,61,62,63].

## 16. Efficacy in GERD Management

Several studies affirm LINX’s ability to significantly reduce GERD symptoms. In a long-term follow-up, Ferrari et al. [57] reported that 88% of patients experienced consistent symptom relief from GERD, with a significant decrease in PPI dependency over six to twelve years. Similarly, Ganz [58] demonstrated that 85% of patients reduced or eliminated PPI use after LINX placement, and quality-of-life scores improved substantially for the majority. This durability in symptom control highlights LINX’s appeal for patients seeking an enduring solution to GERD.

A systematic review by Guidozzi et al. [59] involving a pooled analysis of LINX versus fundoplication found that LINX is not only comparable to fundoplication in symptom control but may also be preferable in terms of preserving physiological functions, such as belching and vomiting. Bell et al. [60,61] also conducted randomized controlled trials comparing LINX with double-dose PPIs, concluding that LINX provided superior control of regurgitation symptoms over medical therapy at the one-year mark. This indicates that LINX is especially beneficial for patients with moderate-to-severe regurgitation that does not respond to medication.

In terms of patient satisfaction, Froiio [62] reviewed LINX’s safety profile across multiple studies and found it favorable overall. They reported that nearly 90% of patients would recommend the procedure to others, reflecting high satisfaction rates. A multicenter trial by Bonavina et al. [63] initially demonstrated the device’s feasibility and safety, with few intraoperative complications and a low rate of adverse events early in the device’s use.

Bell et al. [60] noted that LINX has an advantage in preserving normal physiological functions like vomiting and belching. Unlike fundoplication, which can create a tight wrap that limits these functions, LINX’s flexible design allows patients more freedom, potentially enhancing quality of life and satisfaction.

## 17. Morbidity and Complications

### 17.1. Dysphagia

While effective, LINX is associated with a high incidence of postoperative dysphagia. Studies like Dominguez-Profeta et al. [64] found that around 68% of patients experienced dysphagia immediately after surgery, though this number dropped to about 13% within a year as most cases resolved without intervention. The researchers noted that factors influencing dysphagia include the tightness of the magnetic ring and the patient’s peristaltic reserve, with more beads generally improving outcomes by reducing constriction on the esophagus.

However, persistent dysphagia remains an issue for some patients. In a community hospital study, Czosnyka et al. [65] observed that 17% of patients required endoscopic dilation or even device removal to address severe dysphagia. This highlights the necessity of postoperative monitoring and the need for some patients to undergo further procedures, although most cases resolve over time.

In the study by Froiio et al. [62], several factors impacting the safety and outcomes of LINX magnetic sphincter augmentation for GERD were discussed. Patients with poor esophageal motility were at higher risk of postoperative complications, particularly dysphagia. Since LINX relies on the ability of the esophagus to push food through the augmented sphincter, poor motility can lead to swallowing difficulties and increased pressure on the device.

Proper sizing of the magnetic ring is critical to ensure both effectiveness and safety. A ring that is too tight may exacerbate dysphagia and, in some cases, endoscopic dilation may be required. Rarely, persistent dysphagia could lead to device removal [65].

### 17.2. Risk of Erosion and Migration

Device erosion and migration, though less common than seen before in patients with the Angelchik prosthesis, represent serious complications with LINX.

Salvador et al. [66] and Alicuben et al. [67] presented cases where LINX eroded into the esophageal wall. The incidence of erosion across studies remains low, typically below 1%, yet these cases underscore the importance of careful patient selection, proper sizing of the ring, and follow-up. According to Alicuben [67], device erosion most frequently occurred in patients with pre-existing anatomical challenges or conditions like larger hiatal hernias.

Buckley et al. [68] conducted a study specifically on patients with large hiatal hernias (≥3 cm), finding that while LINX showed favorable results in GERD control, the risk of erosion and migration was heightened in this population. In light of these findings, LINX is often recommended for patients with smaller hernias or no hernia, while those with larger anatomical defects may benefit more from traditional fundoplication or tailored approaches.

Valinoti et al. [69] observed that erosion tends to manifest several months to years postoperatively, underscoring the importance of long-term monitoring. Early signs of erosion may include persistent dysphagia, new or worsening GERD symptoms, or localized pain. However, in some cases, erosion may remain asymptomatic until it becomes severe, requiring intervention.

They underscore the likelihood that more cases of LINX device erosion may emerge with extended follow-up periods. As they suggest, the incidence of erosion might currently appear low, but with longer-term observation, additional cases could surface. They calculated an increasing risk (1.4) of erosions per year of follow-up [69].

This trend is partly attributed to the gradual nature of erosion: even when initially successful, the device’s prolonged contact with esophageal tissue can eventually lead to wear and breakdown of the esophageal lining.

## 18. Electrical Stimulation (EST)

**Figure 3:**
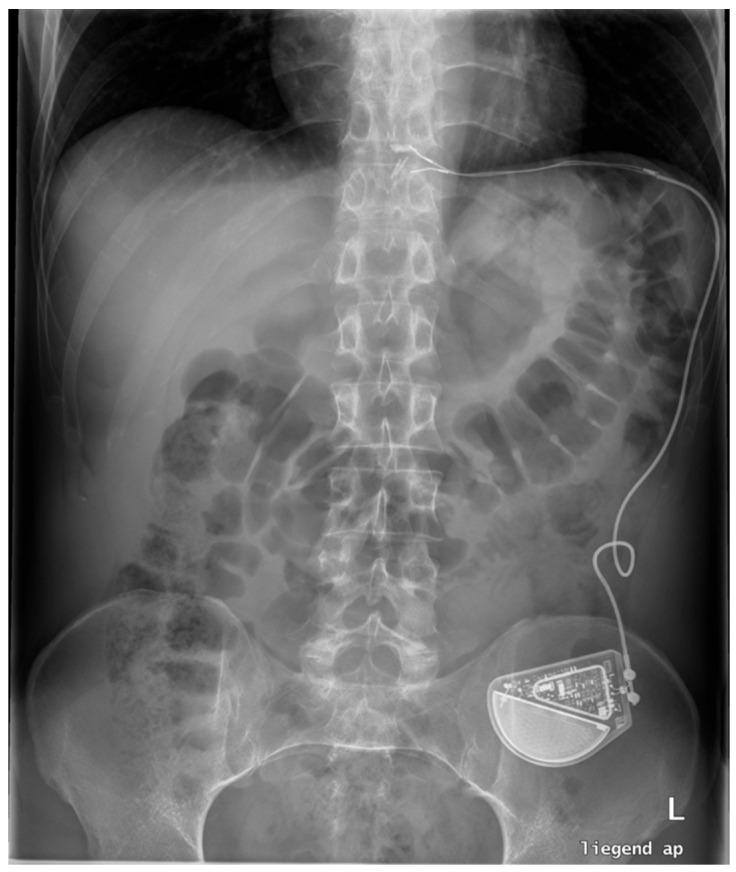
Endostim system with electrodes positioned around the distal esophagus.

EndoStim^®^ (EndoStim BV, Nijmegen, The Netherlands), a novel approach to treat gastroesophageal reflux disease, utilizes electrical stimulation therapy (EST) to strengthen the lower esophageal sphincter (LES) and improve reflux symptoms without altering the anatomical structure [70]. Unlike surgical options, EndoStim^®^ provides a less invasive method to control GERD by using a small, implantable device that delivers controlled electrical impulses via two electrodes that are placed laparoscopically in the distal esophagus near the vagal nerves (Figure 3).

The background research for EndoStim^®^ is based on early studies on electrical stimulation and its effects on the LES, which gradually evolved to show promising therapeutic potential for GERD.

The foundation for EndoStim^®^ began with experimental studies on the effects of electrical stimulation on the esophagus. Ellis et al. [71] demonstrated that applying electrical stimulation to the distal esophagus could prevent reflux in an experimental model. This pioneering work established that electrical currents could influence esophageal and LES function, laying the groundwork for further exploration of EST as a treatment modality.

Decades later, Sanmiguel et al. [72] advanced this research by studying the effects of electrical stimulation on LES pressure in a canine model. Their findings showed that electrical stimulation could reliably increase LES pressure without causing significant side effects, a critical factor for both efficacy and safety. This research confirmed that controlled, targeted stimulation could enhance LES function, suggesting a pathway for clinical applications in humans [72].

Rodríguez et al. [73] conducted a short-term study on electrical stimulation of the LES in GERD patients [73]. The study revealed that electrical stimulation could successfully increase LES pressure, confirming the findings from animal models. Importantly, this study showed that even brief sessions of stimulation could have a measurable impact on LES tone and pressure in human patients, reinforcing the potential of EndoStim^®^ for treating GERD.

Similarily, Banerjee et al. [74] further investigated EST’s effects in GERD patients, observing that electrical stimulation consistently increased LES pressure over time. This study provided some evidence that EST not only strengthens the LES but also maintains enhanced sphincter pressure over extended periods, offering a promising alternative to surgical interventions like fundoplication or device implants.

Results from two studies by Rodriguez et al. demonstrated that this therapy provides substantial symptom relief and sustained improvements in LES function [73,75].

In an open-label prospective trial, 25 GERD patients received EndoStim^®^ therapy. The study found a significant reduction in acid exposure time, with the average percentage of time the esophagus was exposed to acid decreasing from 10.3% at baseline to 5.1% after one year. Symptom relief was significant, with 90% of patients reporting a reduction in GERD symptoms and 64% of patients were able to discontinue or reduce their use of proton pump inhibitors (PPIs). The LES resting pressure also improved, increasing from an average of 7.4 mmHg to 10.2 mmHg, indicating enhanced LES function [73].

A three-year follow-up study by the same working group included 22 patients from the original group [75]. It confirmed sustained improvement, with the average acid exposure time remaining at 5.2%, demonstrating long-term effectiveness in reducing reflux. Overall, 72% of patients continued to experience symptom relief without needing PPIs or only requiring minimal doses. LES resting pressures were maintained, supporting the therapy’s effectiveness in strengthening the LES over the long term [75].

Paireder et al. conducted a study in 17 patients with GERD in whom Endostim^®^ was applied [76]. In contrast to the findings mentioned above, no significant changes could be observed between the baseline and 12 months follow up for the percentage of pH < 4 as well as the LES pressure, as measured by impedance pH measurement and manometry. However, patients reported significant improvement for quality of life evaluation by standardized questionnaires at 12 months follow up [76].

According to the authors, it is difficult to understand how this controversy can be explained, and the possibility of a placebo effect must be taken into account [76].

It must also be mentioned that there is a lack of randomized, sham controlled trials that seek to evaluate the scientific evidence for this approach, as well as the multiple restrictions (e.g., cardiac arrythmia) for its use as pointed out by the manufacturer [70].

## 19. RefluxStop™

**Figure 4:**
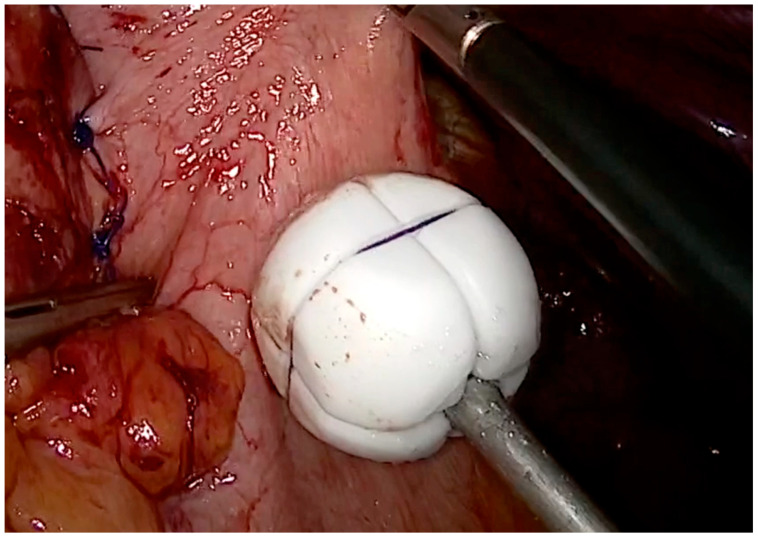
RefluxStop™ applied at the fundus.

RefluxStop™ (RefluxStop™-Implantica, Vduz, Liechtenstein) is a laparoscopically implantable device that blocks the movement of the LES up into the thorax and keeps the angle in its original, anatomically correct position (Figure 4). This new device tries to restore normal anatomy, leaving the food passageway unaffected.

In a prospective multicentric clinical investigation, Bjelovic et al. operated on 50 patients with chronic GERD in order to evaluate the safety of this approach [77]. Secondary outcomes included reduction of total acid exposure time in 24-h pH monitoring and reduction in average daily PPI usage and subject satisfaction.

There were no serious adverse events related to the device. Average GERD-HRQL total score at 1 year improved 86% from baseline (*p* < 0.001). In addition, 24-h pH monitoring compared to baseline showed a mean reduction percentage of overall time with pH < 4 from 16.35 to 0.80% at the 6-month visit (*p* < 0.001), with 98% of subjects showing normal 24-h pH.

At 1-year follow-up, only one subject took regular daily PPIs due to too low placement of the device, thereby prohibiting its function. None or minimal occasional episodes of regurgitation occurred in 97.8% of evaluable subjects. Gas bloating disappeared in 30 subjects and improved in 7 subjects.

The same group recently reported a 4 year follow-up [78], showing that the median GERD-HRQL score was still 90% reduced compared to baseline. Two patients (2/44) used regular daily proton pump inhibitors (PPIs) despite subsequent 24-h pH monitoring of PPI therapy yielding normal results. There were no device-related adverse events (AEs), esophageal dilations, migrations, or explants during the entire study period.

Although promising, these results must be evaluated with caution due to the lack of other controlled studies from other institutions.

## 20. Conclusions

Gastroesophageal reflux disease (GERD) remains a significant global health challenge due to its chronic nature and the limitations and/or controversies of traditional management strategies, such as long-term proton pump inhibitor (PPI) use and classic fundoplication. This review has highlighted the evolution of alternative GERD treatment, showcasing a spectrum of innovative approaches that bridge the gap between conventional pharmacological management and standard surgical approaches.

The advent of minimally invasive techniques, including endoscopic therapies like Stretta, transoral incisionless fundoplication (TIF), and anti-reflux mucosectomy (ARMS), presents an exciting frontier in GERD management. These procedures have been shown to offer the potential to alleviate symptoms, reduce esophageal acid exposure, and decrease dependence on long-term medication. Similarly, laparoscopic advancements aside from the “classical” laparoscopic fundoplication have given some evidence to open new avenues for addressing lower esophageal sphincter dysfunction while preserving physiological functions.

Despite their promise, it is challenging to provide definitive recommendations regarding the adoption of these methods due to several key limitations. First, the lack of strong and consistent scientific evidence supporting the long-term efficacy and safety of many of these therapies leaves unanswered questions about their durability. Second, the potential for late complications, such as device erosion, dysphagia, or anatomical changes, highlights the need for extended follow-up studies. Finally, the limited clinical experience and variability in outcomes associated with some of these techniques underscore the importance of cautious patient selection and ongoing evaluation.

Given these constraints, the importance of individualized treatment strategies cannot be overstated. Multidisciplinary collaboration involving gastroenterologists, surgeons, and informed patient input is critical to navigating this complex therapeutic landscape. While endoscopic and laparoscopic innovations show considerable potential, they should be considered within the context of each patient’s clinical profile, anatomical characteristics, and preferences.

## Figures and Tables

**Table 1:** Approaches for new strategies.

Endoscopic Methods	Radiofrequency (STRETTA)
	Transoral (incisionless) fundoplication
	Endoscopic full thickness plication
	Transoral antegrade suturing
	Mucosal resection/ablation
Laparoscopic methods	Magnetic ring (LINXX)
	Endostim
Combined endoscopic/laparoscopic method	Transoral fundoplication + laparoscopic hiatoplasty
